# Effects of Hyperoxia and Antioxidants on Mortality, Hospital Admissions, and Myocardial Infarction After Noncardiac Surgery: 1‐Year Follow‐Up of a Randomized Controlled Trial

**DOI:** 10.1111/aas.70118

**Published:** 2025-10-07

**Authors:** Frederik C. Loft, Cecilie Holse, Eske K. Aasvang, Morten Vester‐Andersen, Lars S. Rasmussen, Lars N. Jørgensen, Christian S. Meyhoff

**Affiliations:** ^1^ Department of Anaesthesia and Intensive Care Copenhagen University Hospital ‐ Bispebjerg and Frederiksberg Hospital Copenhagen Denmark; ^2^ Novo Nordisk Søborg Denmark; ^3^ Department of Anaesthesia Center of Cancer and Organ Dysfunction, Copenhagen University Hospital ‐ Rigshospitalet Copenhagen Denmark; ^4^ Department of Clinical Medicine University of Copenhagen Copenhagen Denmark; ^5^ Herlev Anaesthesia Critical and Emergency Care Science Unit, Department of Anaesthesiology Copenhagen University Hospital – Herlev and Gentofte Hospital Copenhagen Denmark; ^6^ Personnel Agency Danish Ministry of Defence Copenhagen Denmark; ^7^ Digestive Disease Center Copenhagen University Hospital ‐ Bispebjerg and Frederiksberg Hospital Copenhagen Denmark

## Abstract

**Background:**

Perioperative hyperoxia may be associated with increased long‐term mortality, whereas perioperative antioxidants may be associated with reduced long‐term mortality. This study aimed to determine if high perioperative inspiratory oxygen fraction (FiO_2_) (0.80) compared with normal FiO_2_ (0.30) would increase mortality, hospital admissions, and myocardial infarction (MI) within 1 year after surgery, and whether antioxidants compared with placebo would reduce this.

**Methods:**

This was the preplanned 1‐year follow‐up of 600 patients with cardiovascular risk factors, scheduled for noncardiac surgery. They were randomized in a 2 × 2 factorial design to perioperative FiO_2_ of 0.80 or 0.30 and to receive antioxidants (vitamin C and N‐acetylcysteine) or matching placebo. The primary 1‐year outcome was all‐cause mortality, and secondary 1‐year outcomes were one or more hospital admissions and MIs, respectively. All outcomes were assessed using medical records and analyzed with the Cox proportional hazards model.

**Results:**

Follow‐up was completed for 594 patients (99%). Twenty‐five of 298 patients (8.4%) allocated to FiO_2_ of 0.80 died within 1 year as compared with 17 out of 296 (5.7%) allocated to FiO_2_ of 0.30, HR 1.46 (95% CI, 0.79–2.70), *p* = 0.23. A total of 260 patients had one or more hospital admissions (44%), and seven patients had MI (1.2%) with no significant difference when comparing FiO_2_ of 0.80 with 0.30. Antioxidants had a HR of 0.98 (95% CI, 0.54–1.80), *p* = 0.96 for all‐cause mortality vs. placebo. The interaction between the FiO_2_ and antioxidant administration was statistically significant (*p* = 0.04) with fatalities overrepresented in patients given 80% oxygen and placebo.

**Conclusions:**

Differences in all‐cause mortality, hospital admission, or MI were not statistically significant at 1‐year follow‐up for either oxygen fractions or antioxidant administration in patients undergoing major noncardiac surgery.

**Editorial Comment:**

In this preplanned long‐term study of the VIXIE trial, no differences in total mortality, hospitalization, or myocardial infarction were found for oxygen fractions of 0.80 compared to 0.30 or antioxidant administration compared to placebo. Interestingly, the study showed a higher rate of fatalities with 80% oxygen which appeared only to be present in patients not given the antioxidant intervention, but this is hypothesis‐generating and needs to be further investigated in new clinical trials.

**Trial Registration:** Clinicaltrials.gov identifier: NCT03494387

## 1 Introduction

Patients undergoing general anesthesia receive supplemental oxygen to prevent hypoxia. The World Health Organization (WHO) recommends an inspiratory oxygen fraction (FiO_2_) of 0.80 to prevent surgical site infections [1]. While most studies have found no significant association between high perioperative FiO_2_ and mortality within 30 days [2, 3, 4, 5], an observational study of almost 310,000 patients undergoing major surgical procedures reported a significant association between a high oxygen fraction and postoperative complications, including death [6]. A perioperative FiO_2_ of 0.80 was associated with significantly increased long‐term mortality as compared with FiO_2_ 0.30 in a randomized controlled trial (RCT) including 1382 patients [7]. The excess mortality appeared to be present only in patients undergoing cancer surgery [8]. Observational studies have detected more postoperative cardiac events with increased FiO_2_ [6, 9]. This was not found in trials at short term [10, 11], but the long‐term risk of myocardial infarction (MI) was higher after 80% oxygen, in the aforementioned RCT [12].

Both hyperoxia and the surgical stress response can lead to the generation of reactive oxygen species [13]. When the level of reactive oxygen species exceeds the capacity of antioxidants, oxidative stress occurs, which can cause cell and DNA damage [13, 14]. The VIXIE trial (VitamIn and oXygen Interventions and cardiovascular Events) randomized 600 patients undergoing major noncardiac surgery [2]. Patients were allocated to either a perioperative FiO_2_ of 0.80 or 0.30 and to receiving either antioxidants or matching placebo. No significant differences were found in the primary outcome of the degree of postoperative myocardial injury. The 30‐day mortality was 2.0% in the group of FiO_2_ 0.80% and 1.0% in the 0.30 group. Additional data is required to confirm or refute previous findings of long‐term effects of administering high oxygen fractions perioperatively.

We performed the preplanned follow‐up of all‐cause mortality (primary 1‐year outcome), hospital admissions, and MI from the VIXIE trial with the primary aim of assessing the effect of a perioperative FiO_2_ of 0.80 compared with an FiO_2_ of 0.30 on these outcomes within 1 year.

## 2 Methods

The VIXIE trial protocol and primary results have previously been published [2, 15]. The trial was registered at clinicaltrials.gov (NCT03494387, first posted April 11, 2018). The study was approved by the Danish Medicines Agency (Record No. 2017064658, approval date January 3, 2018), the Regional Research Ethics Committee (Copenhagen, Denmark, Record No. H‐17039073, approval date February 8, 2018), and the Danish Data Protection Agency (Record No. 2012‐58‐0004, approval date January 15, 2018) before initiation. Written informed consent was obtained from all patients before randomization. The trial was conducted in compliance with Good Clinical Practice (GCP) guidelines, monitored by the Danish GCP units and in accordance with the Helsinki Declaration.

The VIXIE trial was a 2 × 2 factorial, blinded, multicenter trial including a total of 600 patients. Patients were included from the Capital Region of Denmark in four centers at three hospitals from April 2018 to January 2020. The trial included patients aged 45 years or older, with cardiovascular risk factors (either stroke, peripheral artery disease, vascular surgery, or two minor risk factors) undergoing major noncardiac surgery in general anesthesia with an estimated surgical duration of more than 1 h. Exclusion criteria were pregnancy, inability to obtain informed consent, preoperative arterial oxygen saturation below 90% without supplementary oxygen, allergy to the interventional drugs, surgery within the last 30 days, or previous treatment with bleomycin. Exclusion criteria for the follow‐up study was withdrawal of consent or cancelation of surgery, and thus the 17 patients excluded from the original trial due to “no data on primary outcome” (i.e., no postoperative high‐sensitive troponin measurement) were included in the modified intention‐to‐treat population of this follow‐up trial.

Patients were randomized by computer‐generated allocation sequence in variable block sizes stratified by center and previous MI or angina. Patients were randomized in a 1:1:1:1 allocation ratio to receive either 0.80 FiO_2_ and antioxidants, 0.80 FiO_2_ and placebo, 0.30 FiO_2_ and antioxidants, or 0.30 FiO_2_ and placebo. The oxygen intervention was initiated immediately after endotracheal intubation and was continued for 2 h postoperatively using identical face masks and flow rates with different oxygen–air mixtures in the postanesthesia care unit [15]. The antioxidant intervention consisted of 3 g vitamin C intravenously prior to anesthetic induction, and an intravenous infusion of 100 mg kg^−1^ N‐acetylcysteine after anesthetic induction, administered over 4 h, or matching placebo of saline.

### 2.1 Outcomes

Data on all‐cause mortality, hospital admissions, and MIs were obtained using the electronic medical record (Epic, Epic Systems Corporation, Verona, Wisconsin, USA) [15]. The primary 1‐year outcome was all‐cause mortality within 1 year. Secondary 1‐year outcomes were one or more hospital admissions of any cause and MIs, respectively. A hospital admission was defined as a new admission lasting 24 h or longer during the follow‐up period. MI was present when a patient received a primary or supplementary diagnostic code of MI during the follow‐up period. The diagnostic codes were assessed according to the International Classification of Diseases version 10 (ICD‐10), using the DI21 to DI219 codes for MI. In case of emigration, the data was censored from the day of emigration. Danish citizens have a personalized civil registration number, used as identification across all hospitals and public authorities. The electronic medical record is connected with the Danish National Hospital Register and the Danish Civil Registration System, which facilitates extraction of vital status and the status of hospital admissions and diagnostic codes of all nonemigrated [16].

### 2.2 Statistical Analyses

For the modified intention‐to‐treat population, time to all‐cause mortality was illustrated with Kaplan–Meier curve and the effects of the oxygen intervention and the antioxidant intervention on the outcomes were assessed using hazard ratios (HR) with 95% confidence intervals (CI) from Cox proportional hazards regression models, both unadjusted and adjusted for center (No. 1, 2, 3, and 4), previous MI/angina (Yes/No), FiO_2_ 0.80 (Yes/No) and antioxidants (Yes/No). We also performed tests of interaction for the interventions and per protocol analyses of patients who received the intended interventions as planned. A *p*‐value of < 0.05 was considered statistically significant. Statistical analyses were performed in SAS Studio 3.8 (SAS Institute Inc., Cary, NC).

## 3 Results

Of the 600 included patients in the VIXIE‐study, two patients withdrew consent, and four patients had surgery canceled, leaving a total of 594 (99%) patients in the modified intention‐to‐treat population for analyses in this 1‐year follow‐up study. Vital status was obtained in all patients, and their characteristics were similar across the study groups (Table 1). Nineteen patients moved to different regions of Denmark during the study period, making it possible to obtain the primary 1‐year outcome of vital status but not hospital admissions or MIs after censoring.

**TABLE 1:** Characteristics of 594 surgical patients randomized to FiO_2_ 0.80 or 0.30 and to antioxidants or placebo.

	FiO_2_ 0.80 (*n* = 298)	FiO_2_ 0.30 (*n* = 296)	Antioxidants (*n* = 299)	Placebo (*n* = 295)
Age, year	72 ± 9	72 ± 9	72 ± 9	72 ± 9
Female sex	121 (40)	124 (42)	129 (43)	116 (39)
Body mass index, kg/m^2^	27 [23, 30]	27 [24, 30]	27 [23–30]	27 [24–31]
American Society of Anesthesiologists physical status				
I	6 (2.0)	2 (0.7)	6 (2.0)	2 (0.7)
II	124 (42)	143 (48)	133 (44)	134 (45)
III	165 (55)	148 (50)	156 (52)	157 (53)
IV	3 (1.0)	3 (1.0)	4 (1.3)	2 (0.7)
Emergent surgery	30 (10)	26 (8.8)	29 (9.7)	27 (9.2)
Previous or current daily smoker	235 (79)	217 (73)	229 (77)	223 (76)
Excessive alcohol consumption^a^	55 (18)	49 (17)	44 (15)	60 (20)
History				
Active cancer	70 (23)	71 (24)	74 (25)	67 (23)
Hypertension	210 (70)	208 (70)	202 (68)	216 (73)
Atrial fibrillation	37 (12)	48 (16)	44 (15)	41 (14)
Coronary artery disease including angina	52 (17)	52 (18)	52 (17)	52 (18)
Myocardial infarction	31 (10)	29 (9.8)	34 (11)	26 (8.8)
Congestive heart failure	17 (5.7)	20 (6.8)	24 (8.0)	13 (4.4)
Pulmonary embolus or deep vein thrombosis	18 (6.0)	14 (4.7)	14 (4.7)	18 (6.1)
Peripheral arterial disease	39 (13)	48 (16)	43 (14)	44 (15)
Chronic obstructive pulmonary disease	38 (13)	41 (14)	40 (13)	39 (13)
Renal failure requiring dialysis or plasma creatinine > 175 μM	8 (2.7)	6 (2.0)	7 (2.3)	7 (2.3)
Diabetes type 1 or 2	63 (21)	55 (19)	58 (19)	60 (20)
Stroke	27 (9.1)	39 (13)	29 (9.7)	37 (13)
Infection within 3 months	19 (6.4)	19 (6.4)	24 (8.0)	14 (4.8)
Other significant noncardiovascular disease	54 (18)	55 (19)	59 (20)	50 (17)
Type of surgery				
Open abdominal surgery	49 (16)	37 (13)	45 (15)	41 (14)
Laparoscopic abdominal surgery	101 (34)	114 (39)	108 (36)	107 (36)
Vascular surgery	60 (20)	64 (22)	61 (20)	63 (21)
Orthopedic surgery	84 (29)	79 (27)	83 (28)	80 (27)
Other	4 (1.3)	2 (0.7)	2 (0.7)	4 (1.4)
Type of anesthesia				
Total intravenous anesthesia	243 (82)	242 (82)	247 (83)	238 (81)
Inhalational	54 (18)	51 (17)	48 (16)	57 (19)
Regional anesthesia	1 (0.3)	3 (1.0)	4 (1.3)	0
Epidural analgesia	44 (15)	45 (15)	34 (11)	55 (19)
Duration of anesthesia, min	200 [105, 406]	210 [96, 397]	200 [152, 277]	209 [157, 272]
Erythrocyte transfusion, mL	490 [245, 500]	490 [250, 980]	485 [245, 735]	520 [490, 980]

*Note:* Data are number (%), mean ± SD, or median [interquartile range].

^a^ > 12 g alcohol/day for women and > 24 g alcohol/day for men.

**FIGURE 1:**
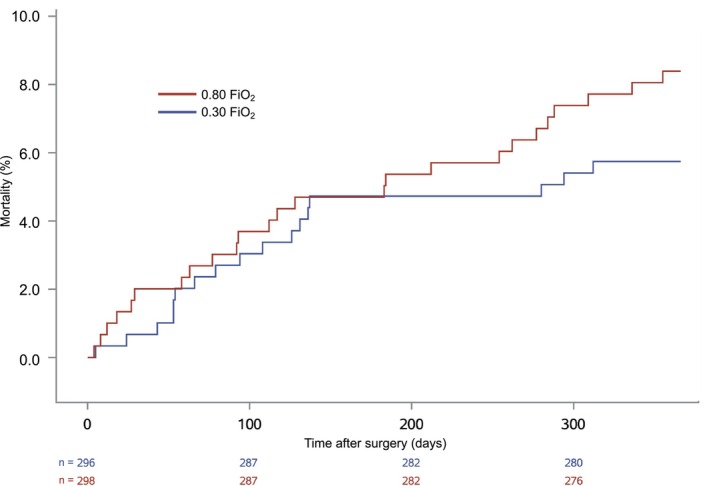
Kaplan–Meier plots for all‐cause mortality of patients randomized to perioperative 0.80 or 0.30 FiO_2_.

**FIGURE 2:**
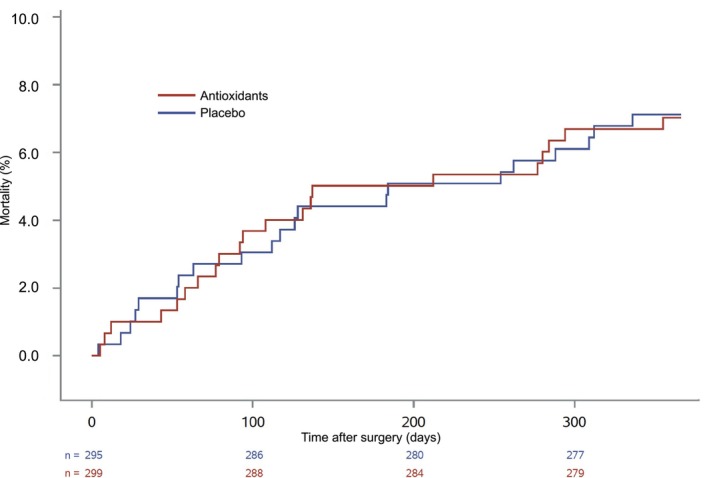
Kaplan–Meier plots for all‐cause mortality of patients randomized to perioperative antioxidants or placebo.

The primary 1‐year outcome of all‐cause mortality within 1 year occurred in 25 of 298 patients (8.4%) allocated to an FiO_2_ of 0.80 as compared with 17 out of 296 (5.7%) allocated to an FiO_2_ 0.30 adjusted HR 1.46 (95% CI, 0.79–2.70), *p* = 0.23 (Figure 1). There was no significant difference in all‐cause mortality between patients given antioxidants vs. placebo (HR 0.98 (95% CI, 0.54–1.80)), *p* = 0.96, with 21 deaths in each group at 1 year (7.1% and 7.0%, respectively) (Figure 2). There were 260 patients with one or more hospital admissions (44%) and the adjusted HRs were 1.02 (95% CI, 0.80–1.30) when comparing FiO_2_ of 0.80 with 0.30 and 0.92 (95% CI, 0.72–1.18) when comparing antioxidants with placebo (Table 2). Seven patients developed MI (1.2%), and the HRs were 1.33 (95% CI, 0.30–5.94) for the oxygen intervention and 0.38 (95% CI, 0.07–1.97) for the antioxidant intervention (Table 2). The unadjusted analyses revealed similar results for both interventions (Table 2).

**TABLE 2:** Effect of perioperative FiO_2_ 0.80 versus 0.30 and antioxidants versus placebo at 1‐year follow‐up.

	FiO_2_ 0.80 (*n* = 298)	FiO_2_ 0.30 (*n* = 296)	0.80 vs. 0.30 FiO_2_ hazards ratio (95% CI)	*p*
All‐cause mortality	25 (8.4)	17 (5.7)		
Unadjusted HR			1.48 (0.80–2.73)	0.22
Adjusted HR			1.46 (0.79–2.70)	0.23
Any hospital admission	131 (44)	129 (44)		
Unadjusted HR			1.02 (0.80–1.30)	0.88
Adjusted HR			1.02 (0.80–1.30)	0.89
Total number of admissions per patient	0.87 [0.0–1.0]	0.85 [0.0–1.0]		
Myocardial infarction	4 (1.3)	3 (1.0)		
Unadjusted HR			1.33 (0.30–5.96)	0.71
Adjusted HR			1.33 (0.30–5.94)	0.71

	Antioxidants (*n* = 299)	Placebo (*n* = 295)	Antioxidants vs. placebo Hazards ratio (95% CI)	*p*
All‐cause mortality	21 (7.1)	21 (7.0)		
Unadjusted HR			0.99 (0.54–1.81)	0.97
Adjusted HR			0.98 (0.54–1.80)	0.96
Any hospital admission	128 (43)	132 (45)		
Unadjusted HR			0.93 (0.73–1.18)	0.55
Adjusted HR			0.92 (0.72–1.18)	0.51
Total number of admissions per patient	0.77 [0.0–1.0]	0.92 [0.0–1.0]		
Myocardial infarction	2 (0.7)	5 (1.7)		
Unadjusted HR			0.39 (0.08–2.01)	0.26
Adjusted HR			0.38 (0.07–1.97)	0.25

*Note:* Hazards ratios are adjusted for center, previous myocardial infarction/angina, and oxygen/antioxidant intervention. Data are number (%), mean [interquartile range], or odds ratio (95% CI).

Abbreviations: FiO_2_, inspired oxygen fraction; HR, Hazards ratio (Cox proportional hazards model).

The per‐protocol population included 554 patients adhering to the intended oxygen intervention and 564 patients that received full antioxidant intervention. The adjusted HR for mortality in the 0.80 FiO_2_ group was 1.50 (95% CI, 0.78–2.89), *p* = 0.22 in the per‐protocol analysis. The remaining analysis did not differ from the results of the modified intention‐to‐treat analysis. Test of interaction between the oxygen interventions and antioxidants/placebo was statistically significant (*p* = 0.036) with fatalities (16 of 42) overrepresented in patients given 80% oxygen and placebo (Table 3).

**TABLE 3:** Distribution of 42 fatalities within 1 year between oxygen and antioxidant interventions.

FiO_2_	FiO_2_ 0.80	FiO_2_ 0.30
Antioxidants	9 (21%)	12 (29%)
Placebo	16 (38%)	5 (12%)

*Note:* The interaction between the FiO_2_ and antioxidant administration was statistically significant (*p* = 0.04) with fatalities overrepresented in patients given 80% oxygen and placebo.

Abbreviation: FiO_2_, inspired oxygen fraction.

## 4 Discussion

In this preplanned long‐term follow‐up of a randomized clinical trial including 600 patients, we found no statistically significant difference in all‐cause mortality, hospital admissions, or MI in patients with cardiovascular risk factors undergoing major noncardiac surgery in general anesthesia, randomized to perioperative high vs. normal FiO_2_ and antioxidants vs. placebo.

Evidence regarding potential effects on mortality with high perioperative oxygen fraction arises from the PROXI trial [3], where patients undergoing acute or elective laparotomy were randomized to an FiO_2_ of 0.80 vs. 0.30 during surgery and for the following 2 h. Follow‐up of 1382 patients after 2.4 years detected a significant difference in mortality with a HR of 1.30 (95% CI 1.03–1.64) [7]. Despite more than a decade between the conduct of the PROXI trial and the present study, the results from PROXI resemble the point estimate of this study's HR of 1.46 for mortality within 1 year in patients allocated to 80% vs. 30% oxygen. Moreover, the Kaplan–Meier curves of these studies are rather similar, as the two groups were closely aligned until approximately day 200, after which mortality increased in the 80% oxygen group.

The results of this study differ from the follow‐ups of two RCTs and a large cluster‐randomized trial. [17, 18] Podolyak and colleagues analyzed two trials with a total of 927 patients, also randomized to an FiO_2_ of 0.80 vs. 0.30, with no significant difference at 4 and 13 years with a follow‐up of 85% [17]. The largest long‐term follow‐up study of mortality was conducted by Jiang and colleagues, who included 2801 patients undergoing 3471 surgical procedures, quasi‐randomized to an FiO_2_ 0.80 or 0.30 using alternating the oxygen intervention in the operating theater [18]. With 69% follow‐up and approximately 13% of patients receiving both interventions, the study detected no significant difference after 3.2 years (HR 0.94, 95% CI, 0.78–1.13).

Some considerations for this discrepancy must be emphasized. In the PROXI trial, the excess mortality appeared to be significant solely in patients undergoing cancer surgery [7, 8]. For those patients that had new or recurrent cancer during follow‐up, this diagnosis was made 100 days earlier in patients that had received an FiO_2_ of 0.80 as compared with 0.30—a finding most prominent in patients with localized cancer at index surgery [8]. The total number of patients with cancer was 451 and 995 in the studies by Podolyak and colleagues and Jiang and colleagues, respectively [17, 18]. None of the studies found any excess mortality in patients with cancer, and the frequency of new or recurrent cancer was also the same in the 80% and 30% oxygen groups in the PROXI trial follow‐up [8].

The current study found a 1‐year mortality of 7%, which is comparable with the finding of Jiang and colleagues but lower than reported in PROXI (approximately 12%–14%). The PROXI trial had a longer follow‐up (2.3 years vs. 1 year), included higher rates of ASA 1 patients (27% vs. 1%), and a higher proportion of emergency surgery (28% vs. 9%) but only laparotomy procedures. Patients in the present cohort were significantly older, with a median age of 72 compared with Jiang and colleagues (54 years) and PROXI (64 years). Despite the relatively low mortality rate, the high reported readmission rate of 44% of the patients in the current study indicates a relevant high‐risk population.

Fonnes and colleagues found a significantly increased risk of MI at a median follow‐up of 3.9 years of the PROXI trial, in contrast to the findings of the current study [12]. Hyperoxia‐induced coronary vasoconstriction has been proposed as a mechanism, but hyperoxia has not been associated with postoperative myocardial injury in trials [2, 10, 11, 19]. In a study by Friess and colleagues, exposure to hyperoxia resulted in a decrease in diastolic function but caused a varying effect on systolic function [20]. They found that patients with good left ventricular myocardial function at normoxia had a decreased systolic function of the left ventricle when exposed to hyperoxia, but patients with reduced function at normoxia had an increase during hyperoxia. The most important mechanism to explore further is, however, the effect of oxygen on cancer angiogenesis, cancer cell migration, and long‐term risk of cancer recurrence [21, 22, 23]. The present study included only 141 patients with active cancer, equally distributed between the intervention groups.

The current study did not find an association between the long‐term outcomes and administration of antioxidants vs. placebo. This contrasts with a previous meta‐analysis showing a potential 26% risk reduction in mortality at the longest follow‐up [24]. However, only four trials included in the meta‐analysis assessed mortality at 1 year or longer [25, 26, 27, 28], and all were small, and either assessed antioxidants in donor grafts prior to transplantation or used a different antioxidant than in this study. None of the four studies found any difference in 1‐year mortality between the groups.

The strength of the current study includes predefined outcomes, rigorous methodology, and almost complete follow‐up, due to the Danish patient registries. Oxygen interventions were given in a standardized manner, which allows the findings to be directly comparable with previous trials. This study also comes with limitations. First, the oxygen intervention was not blinded to anesthetic personnel. Second, although the VIXIE trial was powered to detect differences in myocardial injury within the first few days after surgery, the numbers of fatalities and MIs at 1 year make this study underpowered to exclude clinically important differences, and we did not adjust for multiplicity although this was a secondary analysis after the original publication. Third, the use of diagnostic codes to detect MI may provide some diagnostic challenges, as it requires correct coding by all clinicians, but any bias is unlikely to differ systematically between groups.

The higher rate of fatalities with 80% oxygen appeared only to be present in patients not given the antioxidant intervention. Although the attenuation of reactive oxygen species exposure is a biologically plausible explanation for this, the power of the interaction analysis is limited, and the analysis should be considered exploratory. A significant interaction between FiO_2_ and antioxidants in terms of clinically relevant outcomes has not been described before and needs to be explored further.

In conclusion, no statistically significant difference in all‐cause mortality, hospital admissions, or MI was detected at 1‐year follow‐up in patients undergoing major noncardiac surgery and receiving higher vs. normal FiO_2_ and antioxidants vs. placebo.

## Author Contributions

**Frederik C. Loft:** conceptualisation, data curation, formal analysis, investigation, methodology, project administration and writing – original draft preparation. **Cecilie Holse:** conceptualisation, data curation, investigation, methodology, project administration and writing – review and editing. **Eske K. Aasvang:** conceptualisation, investigation, methodology, project administration, and writing – review and editing. **Morten Vester‐Andersen:** conceptualisation, investigation, methodology, project administration and writing – review and editing. **Lars S. Rasmussen:** conceptualisation, investigation, methodology, project administration and writing – review and editing. **Lars N. Jørgensen:** conceptualisation, investigation, methodology, project administration and writing – review and editing. **Christian S. Meyhoff:** conceptualization, data curation, formal analysis, investigation, methodology, project administration, supervision, and writing – review and editing.

## Conflicts of Interest

C.H. is an employee of Novo Nordisk A/S. C.S.M. and E.K.A. are founders of a start‐up company, WARD24/7 ApS, with the aim of pursuing the regulatory and commercial activities of the WARD‐project (Wireless Assessment of Respiratory and circulatory Distress, a project developing a clinical support system for continuous wireless monitoring of vital signs). WARD24/7 ApS has obtained license agreement for any WARD‐project software and patents. One patent has been filed: ‘Wireless Assessment of Respiratory and circulatory Distress (WARD), EP 21184712.4 and EP 21205557.8’. F.C.L., M.V.A., L.S.R., L.N.J. declare no conflicts of interest. None of the mentioned entities affected the study design, conduct, analysis, or reporting.

## Data Availability

The data that support the findings of this study are available from the corresponding author upon reasonable request and regulatory approval.
